# MiR‐30c protects diabetic nephropathy by suppressing epithelial‐to‐mesenchymal transition in db/db mice

**DOI:** 10.1111/acel.12563

**Published:** 2017-01-27

**Authors:** Yanru Zhao, Zhongwei Yin, Huaping Li, Jiahui Fan, Shenglan Yang, Chen Chen, Dao Wen Wang

**Affiliations:** ^1^ Division of CardiologyDepartment of Internal MedicineTongji HospitalTongji Medical CollegeHuazhong University of Science and TechnologyWuhan430030China; ^2^ Department of CardiologyThe First Affiliated Hospital of Chongqing Medical UniversityChongqing400042China

**Keywords:** diabetic nephropathy, epithelial‐to‐mesenchymal transition, miR‐30c, Snail1, TGF‐β1

## Abstract

Epithelial‐to‐mesenchymal transition (EMT) plays a significant role in tubulointerstitial fibrosis, which is a hallmark of diabetic nephropathy. Thus, identifying the mechanisms of EMT activation could be meaningful. In this study, loss of miR‐30c accompanied with increased EMT was observed in renal tubules of db/db mice and cultured HK2 cells exposed to high glucose. To further explore the roles of miR‐30c in EMT and tubulointerstitial fibrosis, recombinant adeno‐associated viral vector was applied to manipulate the expression of miR‐30c. *In vivo* study showed that overexpression of miR‐30c suppressed EMT, attenuated renal tubulointerstitial fibrosis and reduced proteinuria, serum creatinine, and BUN levels. In addition, Snail1 was identified as a direct target of miR‐30c by Ago2 co‐immunoprecipitation, luciferase reporter, and Western blot assays. Downregulating Snail1 by siRNA reduced high glucose‐induced EMT in HK2 cells, and miR‐30c mimicked the effects. Moreover, miR‐30c inhibited Snail1‐TGF‐β1 axis in tubular epithelial cells undergoing EMT and thereby impeded the release of TGF‐β1; oppositely, knockdown of miR‐30c enhanced the secretion of TGF‐β1 from epitheliums and significantly promoted proliferation of fibroblasts and fibrogenesis of myofibroblasts, aggravated tubulointerstitial fibrosis, and dysfunction of diabetic nephropathy. These results suggest a protective role of miR‐30c against diabetic nephropathy by suppressing EMT via inhibiting Snail1‐TGF‐β1 pathway.

## Introduction

Diabetic nephropathy (DN) is not only a major complication of diabetes, but also a common cause of end‐stage renal disease (ESRD) worldwide. For a long time, most studies focused on glomerular lesions in the progression of DN, and tubular injury has been underestimated (Kanwar *et al*., 2011). Recently, emerging evidences observe that declined renal function is more correlated with tubulointerstitial fibrosis than glomerular fibrosis, and tubular proteinuria may appear prior to microalbumunuria in diabetic patients, suggesting that tubular damage also plays an important role in the pathogenesis of DN (Taft *et al*., 1994; Phillips & Steadman, 2002). Growing evidences demonstrate that inflammation, oxidative stress, and autophagy in tubular epithelial cells (TECs) may be involved in the progression of DN, but the contribution of epithelium dedifferentiation to DN remains poorly understood(Rodriguez‐Iturbe & Garcia Garcia, 2010; Navarro‐Gonzalez *et al*., 2011; Arora & Singh, 2014; Kume & Koya, 2015).

Epithelial‐to‐mesenchymal transition (EMT), a biologic process which epithelial cells transdifferentiate into motile mesenchymal cells, contributes to pathological fibrosis and cancer progression(Kalluri & Weinberg, 2009; Lamouille *et al*., 2014). During EMT, epithelial cells lose apical–basal polarity and junctions, reorganize cytoskeleton, then finally transdifferentiate into mesenchymal cell phenotype, with enhanced migratory and invasive ability, and markedly increased production of extracellular matrix (ECM) components(Kalluri & Neilson, 2003; Thiery *et al*., 2009). Epithelial cells undergoing EMT are found to be involved in fibrosis occurring in organs such as kidney (Liu *et al*., 2016), liver (Kaimori *et al*., 2007), lung (Kim *et al*., 2006), and intestine (Scharl *et al*., 2015). In kidney, blocking EMT of TECs prevented chronic renal injury and fibrosis (Yang & Liu, 2002; Zeisberg *et al*., 2003). More specifically, it has been observed that EMT occurred in TECs of patients with DN, indicated by mesenchymal like α‐smooth muscle actin (α‐SMA)‐positive TECs (Rastaldi *et al*., 2002; Mandache *et al*., 2011). Moreover, in human renal biopsies, the number of mesenchymal marker‐positive TECs was associated with the increased serum creatinine level and the severity of interstitial damage (Rastaldi *et al*., 2002). Together, these data imply an important role of EMT in tubulointerstitial fibrosis of DN, but the mechanisms how EMT activates and contributes to tubulointerstitial fibrosis in diabetes are unclear.

It is widely acknowledged that Snail1 is a master activator of EMT program during development, fibrosis, and cancer (Barrallo‐Gimeno & Nieto, 2005). As a transcription factor, Snail1 not only represses epithelial genes including E‐cadherin by binding to E‐box sequences in the proximal promoter region through their carboxy‐terminal zinc‐finger domains, but also activates mesenchymal phenotype related genes, and finally contributes to EMT (Batlle *et al*., 2000; Cano *et al*., 2000). In kidney, Snail1 was normally expressed during embryonic development and was downregulated upon epithelium differentiation (Boutet *et al*., 2006). Activation of Snail1 in TECs of adult mice is sufficient to induce mesenchymal features in TECs, which is essential for the progress of EMT (Boutet *et al*., 2006). Knockdown of Snail1 in TECs can reduce and even reverse the fibrosis response via inhibition of EMT in fibrotic mouse model (Grande *et al*., 2015; Lovisa *et al*., 2015). These data indicate that Snail1 may be an effective target to block EMT in TECs and thereby protect DN from fibrosis.

MicroRNAs (miRNAs) usually contain 21‐24‐nucleotide (nt) and function as regulating the expression of target mRNAs at the post‐transcriptional level. Multiple studies have demonstrated that miRNAs play key roles in diverse biological processes including EMT. In DN, miR‐21, miR‐30, miR‐34, miR‐192, and miR‐200 have been identified as potential regulators (Kato & Natarajan, 2015). Among these, miR‐30c was proved to prevent EMT in cancers, including renal cell carcinoma, lung cancer, and myeloma (Huang *et al*., 2013; Zhao *et al*., 2014; Zhong *et al*., 2014). In addition, miRNAs sequence profiles of human failed kidney with tubulointerstitial fibrosis and tubular atrophy showed a lower expression of miR‐30c compared to normal biopsies (Ben‐Dov *et al*., 2012). All these findings suggest a possible association of miR‐30c with EMT and DN. We therefore hypothesize that miR‐30c may be involved in the progression of DN by regulating EMT. In this study, we revealed that miR‐30c was a key regulator of EMT in DN by targeting Snail1, and over‐expression of miR‐30c attenuated EMT in TECs as well as the profibrogenic microenvironment, and finally ameliorated renal tubulointerstitial fibrosis and dysfunction in DN.

## Results

### MiR‐30c was downregulated in DN

**Figure 1:**
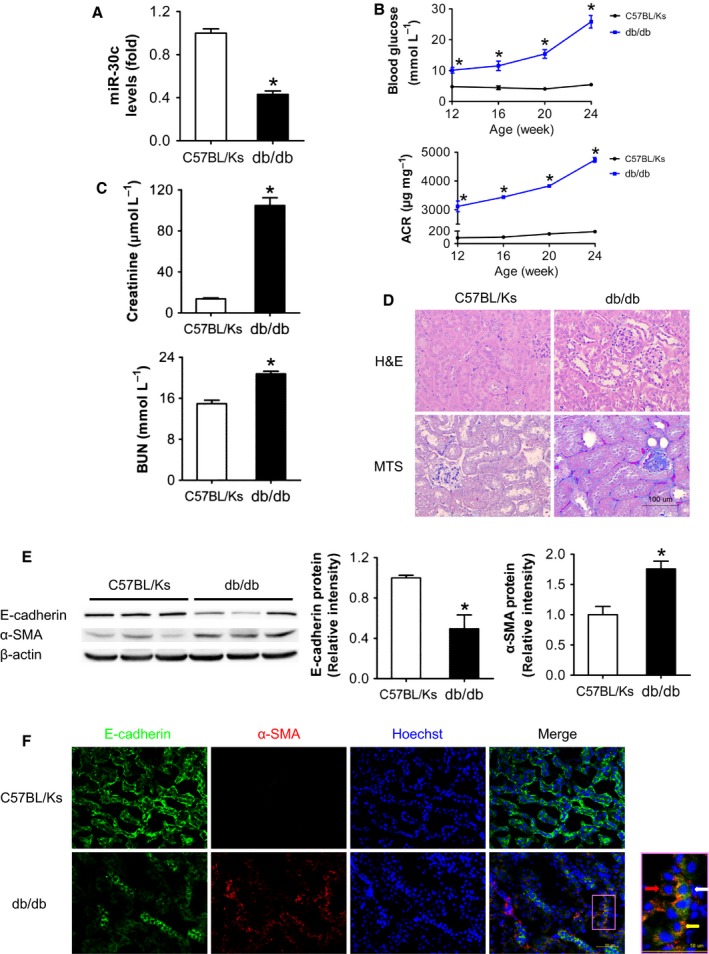
MiR‐30c was decreased, and EMT was induced in db/db mice. (A) Relative miR‐30c expression in renal cortex measured by real‐time PCR. (B) Blood glucose and urine ACR determined every 4 weeks since the age of 12 weeks. (C) Serum creatinine and BUN determined at the age of 24 weeks. (D) Representative images (200×) of H&E and MTS of kidneys from C57BL/Ks and db/db mice. Scale bar, 100 μm. (E) E‐cadherin and α‐SMA protein levels of renal cortex detected by Western blotting. (F) Representative images of immunofluorescence staining for E‐cadherin (green), α‐SMA (red), and Hoechst (blue). Scale bar, 50 μm. Yellow arrow points to E‐cadherin and α‐SMA‐double‐positive tubular epithelial cells (TECs); red arrow points to α‐SMA positive but E‐cadherin‐negative TECs; white arrow points to α‐SMA‐positive myofibroblasts. Data are representative of three experiments. Data are expressed as mean ± SEM, *n* = 8, *P < 0.05 vs. C57BL/Ks.

To explore the role of miR‐30c in DN, expression of miR‐30c in renal cortex of db/db mice with C57BL/Ks background and normal control C57BL/Ks mice were measured by real‐time PCR at 24 weeks. The results showed that miR‐30c was significantly decreased in db/db mice compared with C57BL/Ks in kidney (Fig. 1A).

During the period of observation, blood glucose and urine albumin‐to‐creatinine ratio (ACR) in db/db mice were progressively elevated since the age of 12 weeks (Fig. 1B). At the age of 24 weeks, serum creatinine and blood urea nitrogen (BUN) were significantly increased in db/db mice compared with C57BL/Ks mice (Fig. 1C). Further, hematoxylin–eosin (H&E) and Masson's trichrome staining (MTS) showed that 24‐week‐old db/db mice developed tubulointerstitial lesions with tubular dilation and a severer tubulointerstitial fibrosis (Fig. 1D). Moreover, the protein expression levels of EMT biomarkers (E‐cadherin for epithelial marker and α‐SMA for mesenchymal marker) in renal cortex were detected by Western blots and immunofluorescence assays. The results showed that E‐cadherin was markedly decreased in db/db mice, while α‐SMA was increased (Fig. 1E). Consistently, same results were observed in immunofluorescence assays, and even some tubular epithelial cells (TECs) were also α‐SMA positive, indicating that EMT was induced in db/db mice (Fig. 1F).

These results suggest that db/db mice develop DN and EMT, and miR‐30c might be crucial in pathological process of DN via regulating EMT in TECs.

### Overexpression of miR‐30c attenuated renal dysfunction and EMT in db/db mice

**Figure 2:**
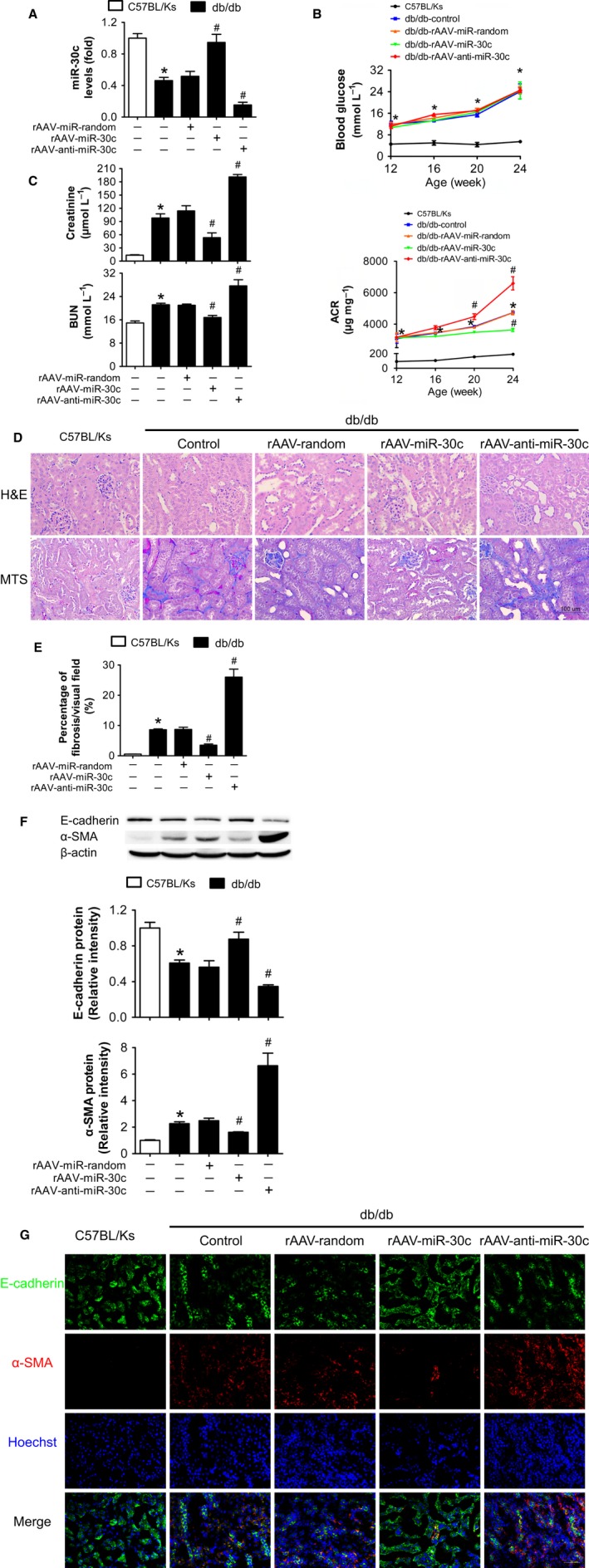
Overexpression of miR‐30c attenuated renal dysfunction and EMT in db/db mice. (A) Relative miR‐30c expression in renal cortex measured by real‐time PCR. (B) Blood glucose and urine ACR determined every 4 weeks. (C) Blood creatinine and BUN determined at the age of 24 weeks. (D) Representative images (200X) of H&E and MTS staining of kidneys from C57BL/Ks and db/db mice with different treatments. Scale bar, 100 μm. (E) MTS quantification as a percentage of overall cortical fibrosis. (F) E‐cadherin and α‐SMA protein levels of renal cortex detected by Western blotting. (G) Representative images of immunofluorescence staining for E‐cadherin (green), α‐SMA (red), and Hoechst (blue). Scale bar, 50 μm. Data are representative of three experiments. Data are expressed as mean ± SEM,* n* = 8, *P < 0.05 vs. C57BL/Ks, ^#^ *P* < 0.05 vs. db/db control.

To investigate the effects of miR‐30c on renal dysfunction in db/db mice, the expression levels of miR‐30c in db/db mice were manipulated by recombinant adeno‐associated viral (rAAV) system. By rAAV‐GFP delivery, we found that most of the kidney cells were efficiently transfected (Fig. S1, Supporting information). After 3 months, it was found that rAAV‐miR‐30c treatment increased miR‐30c expression, while rAAV‐anti‐miR‐30c decreased the expression of miR‐30c in renal cortex of db/db mice (Fig. 2A). Although miR‐30c overexpression had no effect on hyperglycemia development, rAAV‐miR‐30c treatment attenuated urinary protein excretion compared with db/db control mice at the age of 24 weeks (Fig. 2B). Meanwhile, overexpression of miR‐30c significantly decreased serum levels of creatinine and BUN compared with control db/db mice (Fig. 2C). On the contrary, knockdown of miR‐30c by rAAV‐anti‐miR‐30c worsened the renal dysfunction in db/db mice as indicated by further elevated proteinuria, serum creatinine, and BUN (Fig. 2B,C). Moreover, in histological analysis, overexpression of miR‐30c significantly attenuated tubular damage and renal fibrosis (Fig. 2D). Quantification of MTS showed a 40% reduction in renal fibrosis of rAAV‐miR‐30c‐treated group compared to db/db control mice, while nearly threefold fibrosis occurred in rAAV‐anti‐miR‐30c treatment (Fig. 2E). To further investigate the effect of miR‐30c on EMT in TECs, protein levels of E‐cadherin and α‐SMA were detected. Results showed that miR‐30c overexpression reversed the decrease in E‐cadherin and the increase in α‐SMA in db/db mice, while on the contrary, miR‐30c knockdown aggravated these (Fig. 2F). Consistently, rAAV‐miR‐30c treatment reduced the presence of α‐SMA‐positive TECs compared to db/db control mice, suggesting that miR‐30c attenuated EMT in TECs (Fig. 2G). Oppositely, not only the presence of α‐SMA‐positive TECs, but also α‐SMA‐positive myofibroblasts in the interstitium were robustly increased in rAAV‐anti‐miR‐30c‐treated group (Fig. 2G).

All these data suggest that overexpression of miR‐30c attenuates renal dysfunction of DN in db/db mice, while loss of miR‐30c in diabetic kidney causes the exacerbation of EMT in TECs and thereby contributes to tubulointerstitial fibrosis and renal dysfunction.

### Overexpression of miR‐30c alleviated high glucose‐induced EMT in HK2 cells

**Figure 3:**
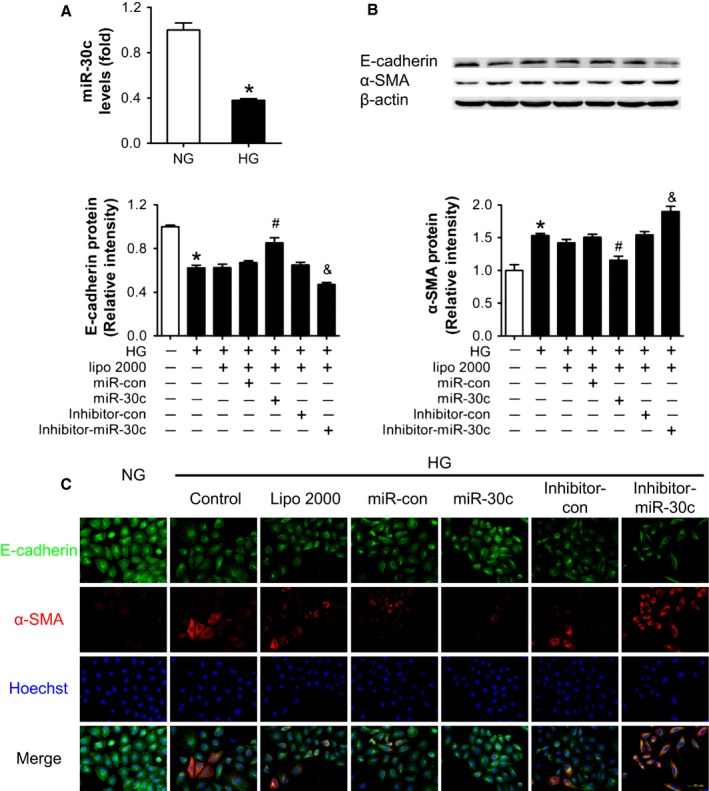
Overexpression of miR‐30c alleviated high glucose‐induced EMT in cultured HK2 cells. (A) Relative miR‐30c expression in cultured HK2 cells exposed to normal glucose (NG, 5 mm) and high glucose (HG, 30 mm) measured by real‐time PCR. (B) E‐cadherin and α‐SMA protein levels of HK2 cells with different treatments detected by Western blot. (C) E‐cadherin and α‐SMA protein levels of HK2 cells with different treatments detected by immunofluorescence staining. Scale bar, 50 μm. Data are representative of three experiments. Data are expressed as mean ± SEM,* n* = 3, *P < 0.05 vs. NG, ^#^ *P* < 0.05 vs. HG + miR‐con, ^&^ *P* < 0.05 vs. HG + inhibitor‐con.

HK2 cells were cultured in medium with high glucose (HG) (30 mm) for 48 h to establish a cell model of hyperglycemia. We found that the level of miR‐30c was decreased in HG‐treated cells compared with normal glucose (NG) (Fig. 3A). On the other hand, the decreased expression of E‐cadherin and increased expression of α‐SMA, indicators of EMT, were induced in HG‐treated HK2 cells (Fig. 3B). Moreover, gain/loss‐of‐function analyses were conducted by transfecting miR‐30c mimics/inhibitor before the treatment of HG. The results showed that overexpression of miR‐30c alleviated the decrease in E‐cadherin and increase in α‐SMA induced by HG, while miR‐30c inhibitor enhanced these effects of HG (Fig. 3B). Consistently, immunofluorescence staining revealed the same results (Fig. 3C). These data suggest that overexpression of miR‐30c protects HK2 cells from HG‐induced EMT.

### Snail1 was a target of miR‐30c

**Figure 4:**
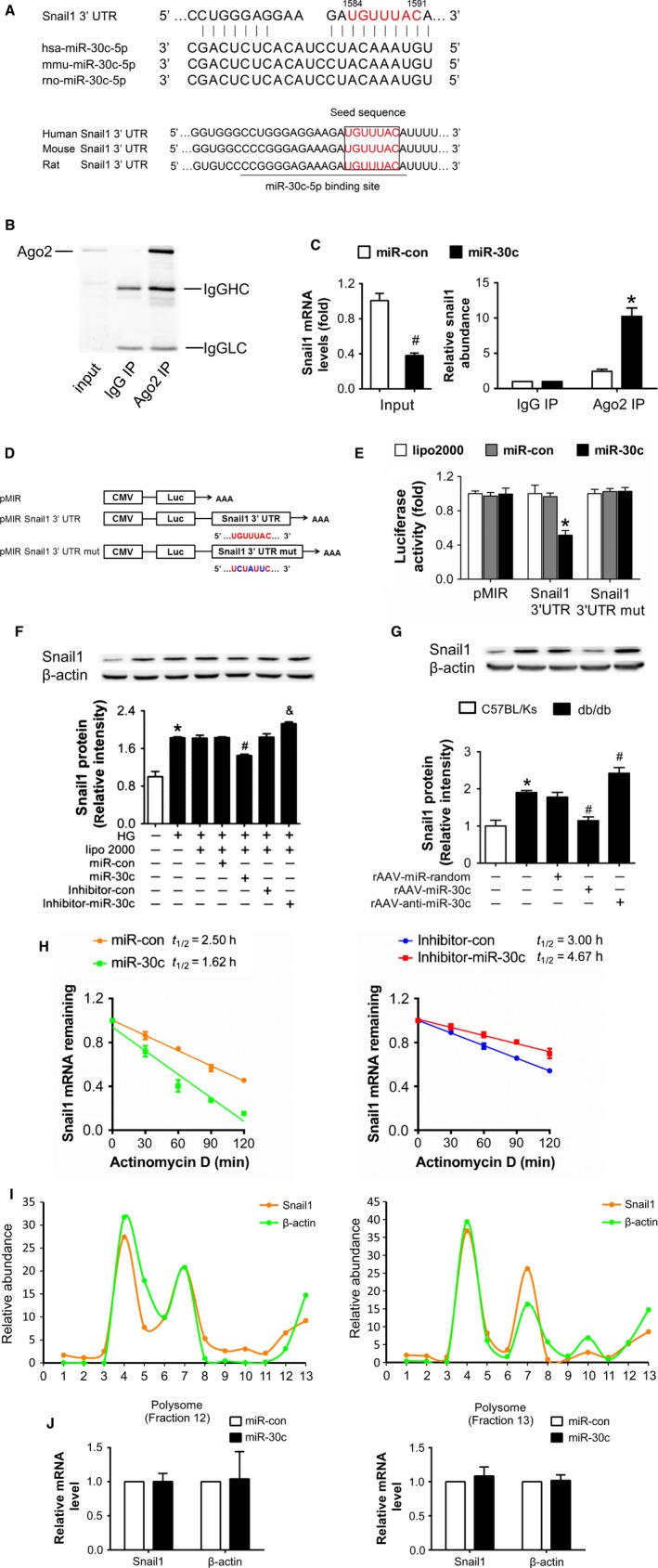
Snail1 is a target of miR‐30c. (A) Sequence alignment between miR‐30c and the 3′‐UTR of Snail1 among several species. (B) Ago2 protein levels in co‐immunoprecipitated products detected by Western blot. IgGHC, IgG heavy chain; IgGLC, IgG light chain. (C) Relative expression of Snail1 in the whole RNA (left) and RNA of the nonspecific IgG or anti‐Ago2 co‐IP (right) from the HG‐treated HK2 cell lysates. ^#^ *P* < 0.05 vs. miR‐con + input, *P < 0.05 vs. miR‐con + IgG IP. (D) Schematic diagram of the luciferase reporter plasmids of pMIR‐Snail1 3′‐UTR and pMIR‐Snail1 3′‐UTR mut, and the potential target site of miR‐30c on the 3′‐UTR of Snail1. (E) Regulation of miR‐30c on 3′‐UTR of Snail1 in HEK293 cells by luciferase reporter assay. *P < 0.05 vs. Snail1 3′‐UTR + miR‐con. (F) Snail1 protein levels of HK2 cells with different treatments detected by Western blot. *P < 0.05 vs. NG, ^#^ *P* < 0.05 vs. HG + miR‐con, ^&^ *P* < 0.05 vs. HG + inhibitor‐con. (G) Snail1 protein levels of renal cortex detected by Western blot. *P < 0.05 vs. C57BL/Ks. ^#^ *P* < 0.05 vs. db/db control. (H) Stability curves of Snail1 mRNA in HG‐treated HK2 cells after transfection of miR‐30c mimics (left) or inhibitor (right). (I) The relative abundance of individual mRNA in each fraction was presented as the percentage of the total fraction following miR‐con (left) or miR‐30c (right) transfection. (J) The association of the Snail1 mRNA with putative polysome fractions (fraction 12 and fraction 13) after miR‐30c mimics transfection. Data are representative of three experiments. Data are expressed as mean ± SEM, *n* ≥ 3.

Using miRNA target prediction programs, we found that Snail1 was one of putative miR‐30c targets and the predicted binding sites were highly conserved during evolution (Fig. 4A). To validate this, Argonaute 2 (Ago2), a crucial component of RNA‐induced silencing complex, was immunoprecipitated from HG‐treated HK2 cell lysates. Analysis of the co‐immunoprecipitated products showed that Ago2 was specifically isolated with the anti‐Ago2 antibody, but not with nonspecific IgG (Fig. 4B). We found that, despite of the lower expression of Snail1 in whole RNA, Ago2 showed increased association with the Snail1 mRNA after miR‐30c transfection (Fig. 4C). We also performed the Ago2 immunoprecipitation in renal cortex of db/db mice with different rAAVs treatments and found that overexpression of miR‐30c enhanced the association between Snail1 mRNA and Ago2 protein *in vivo* (Fig. S2A, Supporting information). Next, we cloned the 3′‐UTR of Snail1 (including wild‐type and seed region mutated sequence) to pMIR‐report vector, respectively, to conduct reporter gene assays (Fig. 4D). Results showed that after co‐transfecting with miR‐30c mimics, the relative luciferase activity of pMIR‐Snail1 3′‐UTR in HEK293 cells was significantly suppressed compared with miR‐con (Fig. 4E). However, this suppressive effect of miR‐30c was abolished by mutating Snail1 3′‐UTR (Fig. 4E). Ago2 co‐IP in cells transfected with reporter plasmids also showed that the reporter mRNA was enriched only in the cell lysates transfected with pMIR‐Snail1 3′‐UTR (Fig. S2B, Supporting information). Furthermore, Western blots showed a higher expression of Snail1 in cultured HK2 cells exposed to HG compared with NG (Fig. 4F). miR‐30c mimics transfection significantly reduced Snail1 level in HG‐treated HK2 cells, while miR‐30c inhibitor further increased Snail1 level (Fig. 4F). Moreover, Snail1 was markedly increased in renal cortex of db/db mice compared with C57BL/Ks (Fig. 4G). And in db/db mice, Snail1 protein level was reduced in rAAV‐miR‐30c‐treated group, while rAAV‐anti‐miR‐30c treatment showed opposite effect (Fig. 4G).

Given that miRNAs typically guide Ago protein complexes to the 3′‐UTR of their target mRNAs leading to its destabilization and/or translation inhibition, we performed the stability assay and polysome analysis. Results of the stability assays demonstrated that Snail1 mRNA was destabilized by miR‐30c treatment, while enhanced stability of Snail1 mRNA was observed following miR‐30c inhibitor transfection (Fig. 4H). Meanwhile, polysome analysis was performed to determine whether miR‐30c might alter the translation of Snail1 mRNA. We characterized the sucrose gradient fraction by showing the distribution of representative small and large ribosomal proteins (RPS3 and RPL4) in the expected fractions, as reported in the literature (Li *et al*., 2016). After RNase I treatment, the putative polysome fractions near the bottom of the gradient (fractions 12 and 13) could be converted to monosome (Fig. S3, Supporting information). Then, the Snail1 and β‐actin mRNA levels in each fraction were detected by real‐time PCR (Fig. 4I). It turned out that both mRNA levels of Snail1 and β‐actin in the putative polysome fractions (fractions 12 and 13) were unaffected by transfection of miR‐30c mimics (Fig. 4J), which suggested that the translation of Snail1 was not inhibited by miR‐30c. Overall, these results suggest that miR‐30c directly inhibits Snail1 expression through binding to its 3′‐UTR and promoting its decay *in vivo* and *in vitro*.

### Downregulation of Snail1 reversed HG‐induced EMT and TGF‐β1secretion in HK2 cells

**Figure 5:**
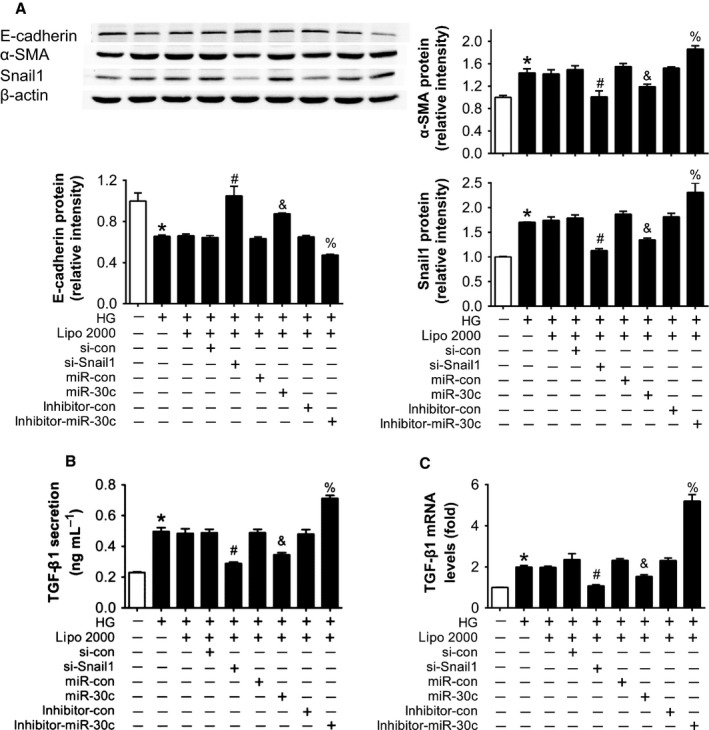
Downregulation of Snail1 reduced high glucose‐induced EMT and TGF‐β1 secretion in cultured HK2 cells. (A) E‐cadherin, α‐SMA, and Snail1 protein levels of HK2 cells with different treatments detected by Western blot. (B) TGF‐β1 level in the culture supernatants measured by ELISA. (C) TGF‐β1 mRNA level in HK2 cells with different treatments detected by real‐time PCR. Data are representative of three experiments. Data are expressed as mean ± SEM,* n* = 3, *P < 0.05 vs. NG, ^#^ *P* < 0.05 vs. HG + si‐con, ^&^ *P* < 0.05 vs. HG + miR‐con, ^%^ *P* < 0.05 vs. HG + inhibitor‐con.

To verify roles of Snail1 in HG‐induced EMT *in vitro*, siRNA against Snail1 was transfected into HK2 cells. Similarly as miR‐30c, knockdown of Snail1 via siRNA increased E‐cadherin expression, but decreased α‐SMA protein level in HG‐treated HK2 cells compared with si‐con transfection (Fig. 5A), suggesting that si‐Snail1 reduces HG‐induced EMT in HK2 cells. Given that in the process of renal fibrosis, TECs could release TGF‐β1 to interstitium (Wang *et al*., 2005), we next investigated the TGF‐β1 secretion of TECs. By ELISA analysis of culture medium, a significant increase in TGF‐β1 secretion was observed in HG‐treated HK2 cells (Fig. 5B). However, the HG‐induced increase in TGF‐β1 in supernatants was significantly inhibited by si‐Snail1 or miR‐30c mimics transfection (Fig. 5B). In contrast, miR‐30c inhibitor aggravated HG‐induced TGF‐β1 secretion (Fig. 5B). The mRNA levels of TGF‐β1 in HK2 cells also showed similar results (Fig. 5C). These data suggest that knockdown of Snail1 reverses EMT and TGF‐β1 secretion induced by HG in cultured HK2 cells.

### MiR‐30c protected DN from fibrosis via reducing TGF‐β1 secretion from TECs undergoing EMT

**Figure 6:**
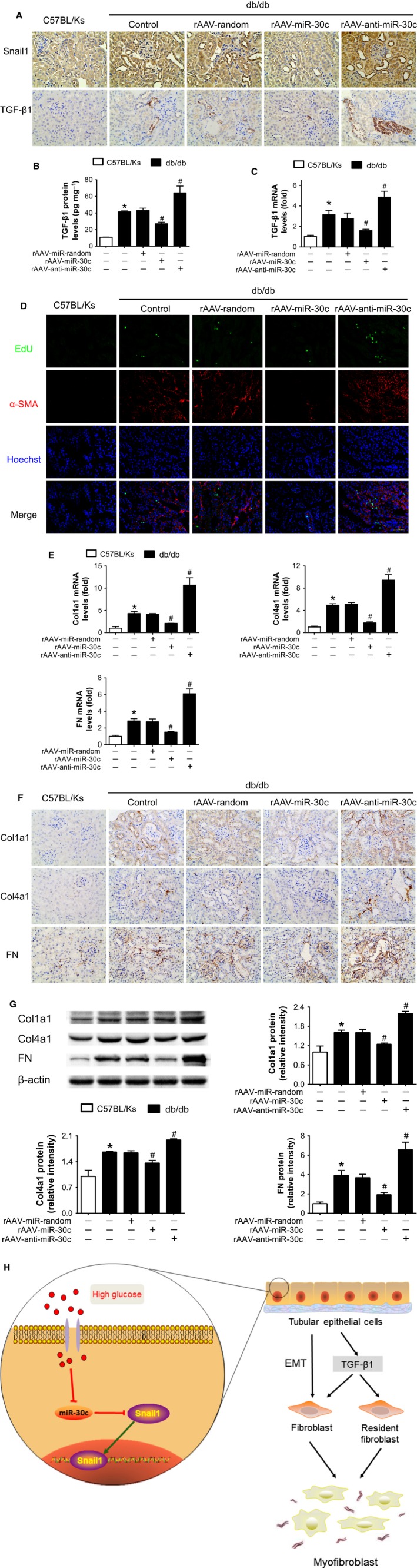
MiR‐30c reduced fibrosis in DN via reducing TGF‐β1 secretion from TECs. (A) Expression levels of Snail1 and TGF‐β1 in renal cortex detected by immunohistochemical staining (400×). Scale bar, 100 μm. (B) TGF‐β1 protein levels in renal cortex lysates measured by ELISA and normalized to total protein concentration in homogenates. (C) Relative TGF‐β1 mRNA level in renal cortex from mice measured by real‐time PCR. (D) Representative images of immunofluorescence staining for EdU (green), α‐SMA (red), and Hoechst (blue). Scale bar, 50 μm. (E) Relative col1a1, col4a1, and FN expression levels in renal cortex from mice measured by real‐time PCR. (F) Relative col1a1, col4a1, and FN expression levels in renal cortex from mice measured by immunohistochemical staining (400×). Scale bar, 100 μm. (G) Relative col1a1, col4a1, and FN expression levels in renal cortex from mice measured by Western blot. Data are representative of three experiments. Data are expressed as mean ± SEM,* n* = 8, *P < 0.05 vs. C57BL/Ks. ^#^ *P* < 0.05 vs. db/db control. (H) Schematic representation of the association among miR‐30c, EMT, and tubulointerstitial fibrosis in DN. In tubular epithelial cells (TECs) of DN, miR‐30c was decreased due to hyperglycemia. The loss of miR‐30c resulted in Snail1 activation, which drove the EMT program in TECs. Snail1‐driven EMT promoted epitheliums to dedifferentiate into fibroblasts. Moreover, TECs released TGF‐β1 to the microenvironment which promoted both the transitional and resident fibroblasts proliferation and activation. Thus, plenty of myofibroblasts accumulated and produced dominant extracellular matrix (ECM) components, contributing to pathologic process of tubulointerstitial fibrosis in DN.

To further verify the role of miR‐30c‐Snail1‐TGF‐β1 axis in mediating renal fibrotic response in db/db mice, the expression levels of Snail1 and TGF‐β1 in kidney of db/db mice with different rAAV treatments were detected. Results showed that Snail1 expression was induced especially in epitheliums of dilated tubular of db/db mice, and overexpression of miR‐30c reduced the Snail1‐positive cells, while miR‐30c knockdown aggravated the effects (Fig. 6A). Consistent with Snail1, TGF‐β1 expression was reduced by miR‐30c proved by immunohistochemistry (Fig. 6A), ELISA (Fig. 6B), and real‐time PCR (Fig. 6C). Given that TGF‐β1 has been identified as a key inducer to activate fibroblasts in DN (Wolf, 2003), EdU incorporation assay was employed to assess the proliferation of fibroblasts and the number of myofibroblasts (activated fibroblasts). Results showed that the number of interstitial EdU‐positive cells, especially α‐SMA‐ and EdU‐double‐positive myofibroblasts, was significantly reduced by miR‐30c overexpression, indicating the proliferation was inhibited (Fig. 6D). On the contrary, the rAAV‐anti‐miR‐30c treatment strongly enhanced the proliferation of interstitial cells (Fig. 6D). Meanwhile, the interstitial α‐SMA‐positive myofibroblast was decreased in rAAV‐miR‐30c‐treated group, but highly increased with rAAV‐anti‐miR‐30c treatment in db/db mice (Fig. 6D). Furthermore, overexpression of miR‐30c inhibited the accumulation of ECM components (Fig. 6E–G). Thus, the Snail1‐TGF‐β1 axis mediated the activation of fibroblasts, fibrogenesis of myofibroblasts, and thereby contributed to tubulointerstitial fibrosis, while miR‐30c prevented this by inhibiting Snail1.

## Discussion

In the present study, we have identified a miR‐30c‐Snail1‐TGF‐β1 fibrosis‐suppressor axis in TECs undergoing EMT. This axis could modulate the activation of fibroblasts and the fibrogenesis of myofibroblasts and finally protect against tubulointerstitial fibrosis in DN (Fig. 6H).

The db/db (C57BLKS/J‐LepR^db/db^) mouse is identified as an obese and diabetic mouse model from C57BLKS/J strain, which is characterized by progressive obese, hyperglycemia and hyperinsulinemia (Hummel *et al*., 1966). Among numerous diabetic mouse models, db/db mouse shows the most consistent and serious elevation of albuminuria and accumulation of ECM components in kidney, which most closely mimics the natural progress of human DN (Sharma *et al*., 2003). Thus, it is widely employed in the research of DN. In our study, we used the db/db mice as animal model of DN and observed progressive increase in blood glucose and proteinuria, poor renal function, and apparent tubulointerstitial fibrosis, which suggested that db/db mice had developed DN.

MiR‐30c has been reported to be decreased not only in heart of patients with diabetic cardiomyopathy, but also in HG‐treated cardiomyocytes, and the loss of miR‐30c mediated prohypertrophic effects of hyperglycemia (Raut *et al*., 2015). Although it was suggested that there was a crucial association between miR‐30c and hyperglycemia in diabetic cardiomyopathy, only few studies focused on miR‐30c and hyperglycemia in DN. In the present study, loss of miR‐30c was observed in both HG‐treated HK2 cells and kidney of db/db mice, which was consistent with the human kidney allografts with tubulointerstitial fibrosis (Ben‐Dov *et al*., 2012). In addition, we found that progressive tubulointerstitial fibrosis and renal dysfunction occurred in DN because of long‐term hyperglycemia exposure. And overexpression of miR‐30c protected against the effects of hyperglycemia in DN, as evidenced by reduced proteinuria, serum creatinine, and BUN. On the contrary, rAAV‐anti‐miR‐30c further reduced miR‐30c in TECs, and thereby the injuries caused by hyperglycemia were highly aggravated. However, increased miR‐30c by rAAV system did not alter the level of blood glucose. As strict glycemic control could reduce the progression of DN (Schernthaner & Schernthaner, 2013), our study suggested that miR‐30c played a protective role in DN independent of the benefits from reduced blood glucose.

Then, we verified Snail1 was a direct target of miR‐30c. Snail1, a zinc‐finger transcription factor, is suppressed during renal development and remains silent in the adult kidney (Boutet *et al*., 2006). The loss of miR‐30c in TECs exposed to hyperglycemia resulted in pathological activation of Snail1, which drove the program of EMT. Following Snail1 activation, we observed that EMT occurred in renal tubules of db/db mice, indicated by decreased epithelial marker (E‐cadherin) but increased mesenchymal marker (α‐SMA) in TECs. Moreover, we found the renal function was inversely correlated with the percentage of Snail1 or α‐SMA‐positive TECs, which are undergoing EMT, as well as the severity of renal tubulointerstitial fibrosis. This is consistent with an earlier observation, which reported that the number of TECs with EMT features in human renal biopsies was associated with serum creatinine level and the degree of interstitial damage (Rastaldi *et al*., 2002). Overexpression of miR‐30c inhibited the increased Snail1 expression induced by hyperglycemia, therefore suppressed EMT and alleviated renal tubulointerstitial fibrosis and dysfunction in DN. In contrary, knockdown of miR‐30c further enhanced hyperglycemia‐induced Snail1 expression, followed by severer EMT and damages in DN. Therefore, our data suggest that the miR‐30c‐Snail1 axis plays a protective role via suppressing hyperglycemia‐induced EMT in DN.

Moreover, Snail1 has been reported to activate the TGF‐β pathway in breast cancer (Dhasarathy *et al*., 2011). TGF‐β1 expression and activation in renal fibrosis were dependent on Snail1 activation in TECs (Grande *et al*., 2015). As expected, we observed that hyperglycemia‐induced Snail1 activated TGF‐β1 expression in TECs of DN *in vivo*. Consistently, exposure to HG increased TGF‐β1 secretion to supernatants *in vitro*. Besides, knockdown of Snail1 by Snail1 siRNA or miR‐30c mimics reduced TGF‐β1 release from HG‐treated HK2 cells. Thus, miR‐30c‐Snail1 axis inhibited hyperglycemia‐induced TGF‐β1 secretion in DN.

In the progression of renal fibrosis, the presence of myofibroblasts (activated fibroblasts) is essential for ECM components formation and their numbers may associate with renal function outcomes (Eddy, 2014). Myofibroblasts are defined as interstitial cells with a feature of fibroblastic morphology and expression of myocyte markers, such as α‐SMA (Strutz & Zeisberg, 2006). Lineage tracing showed that except EMT, local proliferation of resident fibroblasts was also an important cellular origin of myofibroblasts (LeBleu *et al*., 2013). Furthermore, the proliferation and activation of fibroblasts depend on profibrogenic cytokines, such as TGF‐β1 (Grande & Lopez‐Novoa, 2009). Then, TGF‐β1 released by TECs exposed to hyperglycemia promoted the generation of myofibroblasts. In the present study, we assessed the functions of miR‐30c‐Snail1‐TGF‐β1 axis *in vivo* and *in vitro*. Overexpression of miR‐30c inhibited Snail1‐TGF‐β1 and thereby suppressed proliferation of fibroblasts, indicated by less EdU‐positive interstitial cells, as well as less fibrogenesis of myofibroblasts. Oppositely, rAAV‐anti‐miR‐30c treatment enhanced the hyperglycemia effects on tubulointerstitial fibrosis via aggravating Snail1‐TGF‐β1 axis. These together suggested that the miR‐30c‐Snail1 axis played a protective role via suppressing hyperglycemia‐induced TGF‐β1 release from TECs in DN.

Conclusively, our data provide novel evidences that the TECs undergoing EMT play important roles in progression of tubulointerstitial fibrosis in DN. MiR‐30c directly targeted Snail1 in TECs, then suppressed EMT and TGF‐β1 release, and thereby inhibited hyperglycemia‐induced tubulointerstitial fibrosis in DN. These findings suggested a potential target for promising therapeutic intervention in DN.

## Experimental procedures

### Reagents

DMEM/F12, DMEM, and fetal bovine serum (FBS) were obtained from GIBCO (Grand Island, NY, USA). Lipofectamine 2000 (Lipo 2000) reagent was from Invitrogen (Life Technologies Corporation, Carlsbad, CA, USA). The primers of miR‐30c and U6 real‐time PCR, miR‐30c mimics, miR‐30c inhibitor, Snail1 siRNA, and their controls were purchased by RiboBio (Guangzhou, China). The primers of mRNA real‐time PCR were synthesized by BGI Tech (Shenzhen, China). Antibodies against E‐cadherin (Cat No: A3044), α‐SMA (Cat No: A2625), Snail1 (Cat No: A5544), RPS3 (Cat No: A2533), RPL4 (Cat No: A5886), col1a1 (Cat No: A1352), col4a1 (Cat No: A10710), FN (Cat No: A0966), and TGF‐β1 (Cat No: A2124) were purchased from ABclonal Biotech (Cambridge, MA, USA). Anti‐β‐actin (Cat No: sc‐47778) was from Santa Cruz Biotech (Santa Cruz, CA, USA). Anti‐Ago2 (Cat No: H00027161‐M01) was from Novus Biologicals. Prestained protein markers were from Fermentas (Thermo Fisher Scientific Inc., Rockford, IL, USA). Polyvinylidene difluoride (PVDF) membranes were from Millipore (Merck KGaA, Darmstadt, Germany). Horseradish peroxidase‐conjugated secondary antibodies and enhanced chemiluminescence reagents were from Pierce Biotechnology (Thermo Scientific). Alexa Fluor^®^ 488 Donkey Anti‐Rabbit IgG (H+L) Antibody (Cat No: A‐21206) and Alexa Fluor^®^ 594 Donkey Anti‐Mouse IgG (H+L) Antibody (Cat No: A‐21203) were from MOLECULAR PROBES (Thermo Scientific). Other reagents were purchased from Sigma‐Aldrich Company unless otherwise specified.

### Preparation and construction of recombinant adeno‐associated virus (rAAV)

To manipulate the expression of miR‐30c *in vivo*, the rAAV (type 9) was employed. The rAAV system (type 9) was a kind gift from Dr. Xiao Xiao (University of North Carolina at Chapel Hill). For the expression of miR‐random, miR‐30c, and anti‐miR‐30c, oligonucleotides were designed as miR‐random (5′‐GATCCTTTGTACTACACAAAAGTACTGTTCAAGAGACAGTACTTTTGTGTAGTACAAACCGC‐3′), miR‐30c (5′‐GATCC TGTAAACATCCTACACTCTCAGCTTCAAGAGAGCTGAGAGTGTAGGATGTTTACACCGC‐3′), anti‐miR‐30c (5′‐GATCCGCTGAGAGTGTAGGATGTTTACATTCAAGAGATGTAAACATCCTACACTCTCAGCCCGC‐3′) according to the mature sequence of hsa‐miR‐30c‐5p provided by miRBase (Accession: MIMAT0000244). The sequence of miR‐random was provided by RiboBio. The rAAVs were packaged by triple plasmids co‐transfection in HEK293 cells and purified as described previously (Jiang *et al*., 2007). The resultant rAAVs were assigned as rAAV‐miR‐random, rAAV‐miR‐30c, and rAAV‐anti‐miR‐30c, respectively.

### Animals

All animal experiments were approved by the Institutional Animal Research Committee of Tongji Medical College and complied with standards stated in the NIH Guidelines for the Care and Use of Laboratory Animals. Male db/db mice on C57BL/Ks background and control C57BL/Ks mice were purchased from Model Animal Research Center of Nanjing University (Nanjing, China). All the mice were maintained with 12‐h light/12‐h dark photoperiods with free access to water and food. Db/db mice were randomly divided into four groups (control, rAAV‐miR‐random, rAAV‐miR‐30c, and rAAV‐anti‐miR‐30c, *n* = 8 for each group), and they were treated with corresponding rAAVs via tail vein injection at the age of 12 weeks. Then, anaesthetization was preformed with intraperitoneal injections of a xylazine (5 mg kg^−1^) and ketamine (80 mg kg^−1^) mixture, placed in a supine position before mice were sacrificed at the age of 24 weeks. Tissue samples were collected for paraffin embedding or snap‐frozen in liquid nitrogen and stored at −80 °C later.

### Blood and urine examination

After mice were fasted overnight, blood glucose level was measured by Glucose LiquiColor^®^ Test (Stanbio Laboratory, Boerne, TX, USA) every 4 weeks. Twenty‐four‐hour urine was collected by metabolic cage every 4 weeks. Serum creatinine, BUN, and urinary creatinine were determined on an AEROSET Clinical Chemistry System (Abbott Laboratories, Chicago, IL, USA). Urine albumin concentration was determined by the mouse albumin ELISA kit (Bethyl Laboratories, Montgomery, TX, USA).

### Histology and immunohistochemical staining

Kidney tissues fixed in formalin were paraffin embedded and cut into 4‐mm‐thick sections and stained with hematoxylin–eosin (H&E) and Masson trichrome staining (MTS). Images were acquired by light microscope, and MTS was quantified using image pro‐plus Software 6.0 (Media Cybernetics, Bethesda, MD, USA).

For immunohistochemistry of paraffin‐embedded tissue, deparaffinized and rehydrated sections went through microwave‐based antigen retrieval, followed by quenching in 1% hydrogen peroxide solution for 15 min. After blocking with 5% donkey serum blocking buffer for 1 h and staining overnight with antibodies against Snail1, TGF‐β1, FN, col1a1, or col4a1 (dilution 1:200), respectively, the sections were further incubated with peroxidase‐conjugated secondary antibodies and DAB, then counterstained with hematoxylin. Images were acquired by light microscope (400×).

For immunofluorescence of frozen tissue, OCT‐embedded frozen tissue sections (6 μm) were fixed in cold acetone, while HK2 cells on the bottom layer were fixed in 4% paraformaldehyde and blocked with 5% donkey serum blocking buffer for 1 h. After staining overnight with antibodies against E‐cadherin or α‐SMA (dilution 1:100), respectively, the sections and cells were further incubated with Alexa Fluor^®^ secondary antibodies for 1 h and then counterstained with Hoechst 33342 (RiboBio). Sections and cells were observed under the confocal microscope (Olympus, FV500‐IX71, Tokyo, Japan).

### Cell culture, transfection, and treatment

HK2 and HEK293 cells were from American Type Tissue Collection and were cultured in DMEM/F12 or DMEM supplemented with 10% FBS, respectively. Cells were grown at 37°C with a 95% air, 5% CO_2_ atmosphere. Cells were transfected with miR‐30c mimics (100 nm, similarly hereinafter), miR‐30c inhibitor (100 nm), siRNA against human Snail1 (100 nm), or their negative control (100 nm), respectively, using Lipo 2000 reagent according the manufacturer's protocol. After transfection, cells were incubated with normal (5 mm) or high (30 mm) glucose for 48 h and then collected.

### RNA isolation and detection

Total RNA was collected from frozen tissues or cells by TRIzol Reagent (Invitrogen, Carlsbad, CA, USA) according to the manufacturer's protocol. Total RNA (2 μg) was reverse transcribed using the first‐strand cDNA synthesis kit (Thermo Scientific). The primers of miRNA or mRNA and Maxima SYBR Green/ROX qPCR Master Mix (Thermo Scientific) were used for real‐time PCR to detect the relative quantification of RNA according to the manufacturer's protocol with the 7900HT Fast Real‐Time PCR system (Applied Biosystems, Foster City, CA, USA). Each sample has triplicate duplication measurements. U6 small nuclear RNA was used as endogenous control to miRNA. β‐actin was used as endogenous control to mRNA.

### Western blot

Protein samples from cell and mice kidney lysates (30 μg) were separated by SDS‐PAGE electrophoresis using a 10% (wt/vol) acrylamide gel and were transferred to a PVDF membrane. After incubation with primary and secondary antibodies, the bands were visualized by enhanced chemiluminescence kit. The intensities of individual bands were analyzed by densitometry using imagej (National Institutes of Health Software, Bethesda, MD, USA) and normalized to the β‐actin level.

### Target prediction of miRNA

The bioinformatic prediction web sites miRBase (http://www.mirbase.org/), TargetScan (http://www.targetscan.org/) and RNAhybrid (http://bibiserv.techfak.uni-bielefeld.de/rnahybrid/) were applied for miR‐30c target prediction.

### Co‐immunoprecipitation with anti‐Ago2 antibody

Twenty‐four hours after transfection with miR‐30c mimics or miR‐con, HG‐treated HK2 cells were lysed and then immunoprecipitated with anti‐Ago2 antibody or IgG (Santa Cruz Biotech) using protein G Sepharose beads (Santa Cruz Biotech), as described previously (Beitzinger & Meister, 2011; Li *et al*., 2016; Yin *et al*., 2016). After washing, a small aliquot of beads was transferred to a new tube for Western blot using anti‐Ago2 antibody to confirm efficient precipitation of Ago protein complexes. The remaining products were extracted with TRIzol, and the levels of Snail1 mRNA were quantified by real‐time PCR. Lysates of renal cortex of db/db mice with different rAAVs treatments were also analyzed.

### Dual luciferase assay

For dual luciferase assay, 400 ng of pMIR‐Snail1 3′‐UTR, pMIR‐Snail1 3′‐UTR mutant, or the empty vector was transfected into HEK293 cells with 40 ng of pRL‐TK plasmid (Promega, Madison, WI, USA), respectively. Meanwhile, miR‐30c mimics or miR‐con was co‐transfected with those reporter plasmids at a final concentration of 100 nm. Forty‐eight hours later, luciferase activity was detected by Dual‐Luciferase Reporter Assay System (Promega) according to the manufacturer's protocol. Renilla luciferase activity was used to normalize the transfection efficiency.

### mRNA stability

mRNA stability assays were performed as previously reported (Phatak *et al*., 2016). Twenty‐four hours after transfection, HG‐treated HK2 cells were exposed to medium containing Actinomycin D (Sigma‐Aldrich, St. Louis, MO, USA) at a final concentration of 0.5 μg mL^−1^. Cells were harvested at 0, 30, 60, 90, and 120 min, respectively. Total RNA was isolated from each sample, and real‐time PCR was performed in triplicate as described above. The half‐life was calculated from the first order equation *t*_1/2_ = ln2/k.

### Polysome analysis

The polysome analysis was performed as described previously (Tiedje *et al*., 2012; Li *et al*., 2016). Briefly, HK2 cells were treated with cycloheximide and lysed. The ribosome extracts (400 μL each) were loaded on a 10–50% sucrose gradient and centrifuged at 180 000 *g* for 260 min in a SW40.1 Ti Rotor (Beckman Coulter, Fullerton, CA, USA). Subsequently, 13 gradient fractions were collected for RNA and protein analysis. Ribosomal proteins (RPS3 and RPL4) on individual gradient fractions were detected by Western blotting, and specific mRNA transcripts were quantified by real‐time PCR. The assignment of putative polysomes was based on the distribution of ribosomal proteins. To characterize putative polysomes, the lysate was treated with 5 U mL^−1^ RNase I for 40 min at 25 °C to convert polysome to monosome. The relative abundance of individual transcripts in each fraction was presented as the percentage of the total fraction.

### Enzyme‐linked immunosorbent assay (ELISA)

HK2 cells were cultured with different treatment for 48 h, and then, the supernatants were collected. The TGF‐β1 was quantified using a kit from R&D Systems (Minneapolis, MN, USA) according to the manufacturer's protocol.

### EdU incorporation assay

EdU (50 mg kg^−1^, RiboBio) was subcutaneously injected to mice every day for 3 days before sacrifice as described previously (Liu *et al*., 2015). Then EdU staining was performed according to manufacturer's instructions (RiboBio). Sections were observed under the confocal microscope (FV500‐IX71; Olympus).

### Statistics

Data are expressed as mean ± SEM. The Student's *t*‐test and ANOVA were performed among different groups. All calculations were performed by spss 17.0 software (IBM Software, Chicago, IL, USA), and differences with *P* < 0.05 were considered significant.

## Funding

This work was supported by grant from the National Natural Science Foundation of China (No. 91439203, 31571197 and 31400997). The funders had no role in study design, data collection and analysis, decision to publish, or preparation of the manuscript.

## Author contributions

Y. Z. designed and performed the experiments, analyzed the data, and wrote the manuscript; Z. Y., H. L., J. F., and S. Y. participated in performing the experiments; C. C. and D. W. W. designed the experiments and wrote the manuscript.

## Conflict of interest

There is no potential conflict of interest.

## Supporting information


**Fig. S1** Representative images of GFP staining in kidney.
**Fig. S2** Ago2 IP in renal cortex of db/db mice and HK2 cells transfected with reporter plasmids.
**Fig. S3** The distribution of ribosomal proteins in polysome analysis.
