# Supplementary material for: MiR‐30c protects diabetic nephropathy by suppressing epithelial‐to‐mesenchymal transition in db/db mice
Source: Aging Cell. 2017 Jan 27;16(2):387–400. doi: 10.1111/acel.12563 (PMC5334541; doi:10.1111/acel.12563)
Supplement: Supplementary file 1 — Fig. S1 Representative images of GFP staining in kidney. Fig. S2 Ago2 IP in renal cortex of db/db mice and HK2 cells transfected with reporter plasmids. Fig. S3 The distribution of ribosomal proteins in polysome analysis. [file ACEL-16-387-s001.doc]

**MiR-30c protects diabetic nephropathy** **by** **suppressing** **epithelial-to-mesenchymal transition in db/db mice**

Yanru Zhao^1^, M.D.; Zhongwei Yin^1^, M.D.; Huaping Li^1^, M.D.; Jiahui Fan^1^, M.D.; Shenglan Yang^2^, M.D., Ph.D.;Chen Chen^1^, M.D., Ph.D.; and Dao Wen Wang^1^, M.D., Ph.D.

^1^Division of Cardiology, Department of Internal Medicine, Tongji Hospital, Tongji Medical College, Huazhong University of Science and Technology, Wuhan 430030, China; ^2^Department of Cardiology, The First Affiliated Hospital of Chongqing Medical University, Chongqing 400042, China.

Corresponding authors:

Chen Chen and Dao Wen Wang

Division of Cardiology, Department of Internal Medicine, Tongji Hospital, Tongji Medical College, Huazhong University of Science & Technology

1095# Jiefang Ave., Wuhan 430030, China

Tel. & Fax: 86-27-8366-3280

Email: [chenchen@tjh.tjmu.edu.cn](mailto:chenchen@tjh.tjmu.edu.cn); [dwwang@tjh.tjmu.edu.cn](mailto:dwwang@tjh.tjmu.edu.cn)

**Running title:** miR-30c and diabetic nephropathy

**Keyword:** diabetic nephropathy; miR-30c; epithelial-to-mesenchymal transition; Snail1; TGF-β1

**Supplemental Figure 1**


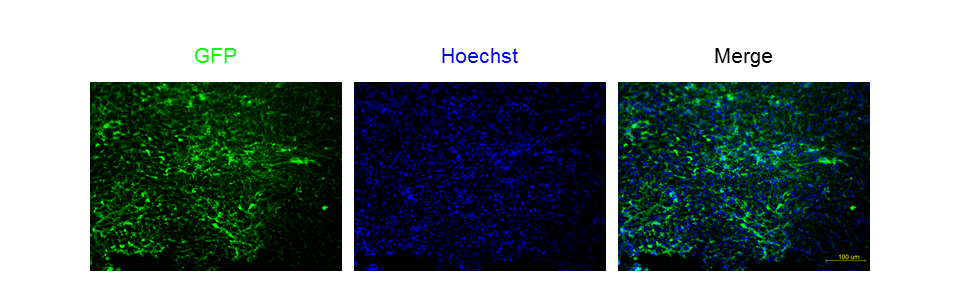


Supplemental Figure 1. Representative images of GFP staining in kidney. Immunofluorescence staining for GFP (green) and Hoechst (blue) in kidney of C57BL/Ks mice after rAAV-GFP injection. The efficiency of infection was presented as the percentage of GFP-positive cells in total cells in kidney. Scale bar, 100um.

**Supplemental Figure 2**


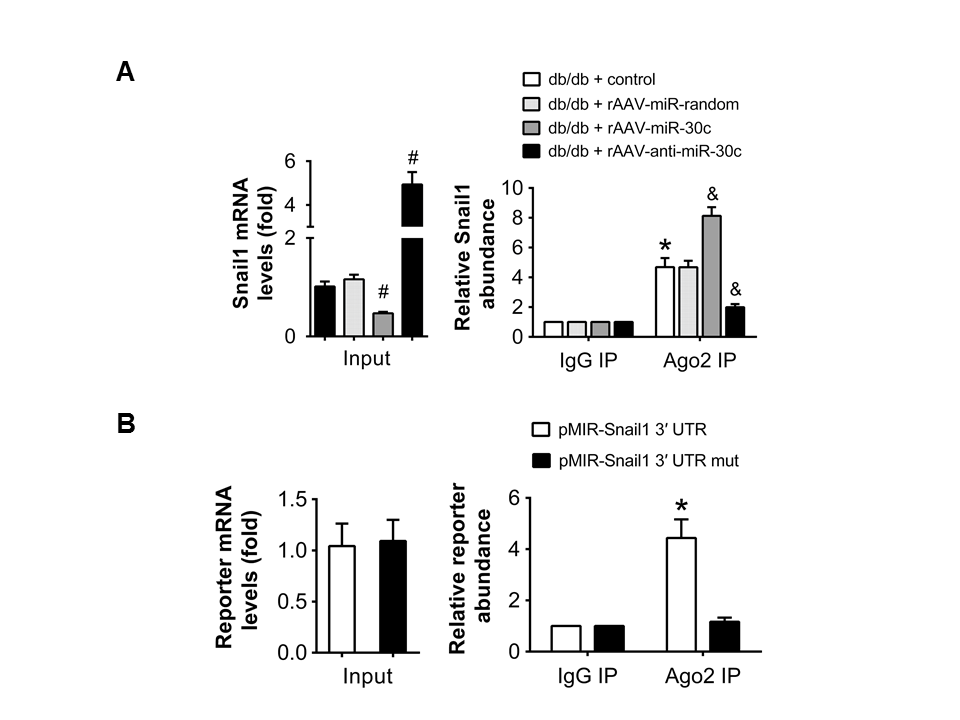


Supplemental Figure 2. Ago2 IP in renal cortex of db/db mice and HK2 cells transfected with reporter plasmids. (A) Relative expression of Snail1 in the whole RNA (left) and RNA of the nonspecific IgG or anti-Ago2 co-IP (right) from the renal cortex of db/db mice. ^#^P<0.05 versus db/db control + input, *P<0.05 versus db/db control + IgG IP. ^&^P<0.05 versus db/db control + Ago2 IP. (B) Relative expression of reporter in the whole RNA (left) and RNA of the nonspecific IgG or anti-Ago2 co-IP (right) from the HK2 cell lysates transfected with pMIR-Snail1 3′ UTR or pMIR-Snail1 3′ UTR plasmids. *P<0.05 versus pMIR-Snail1 3′ UTR + IgG IP. Data are representative of three experiments. Data are expressed as mean ± SEM, n≥3.

**Supplemental Figure 3**


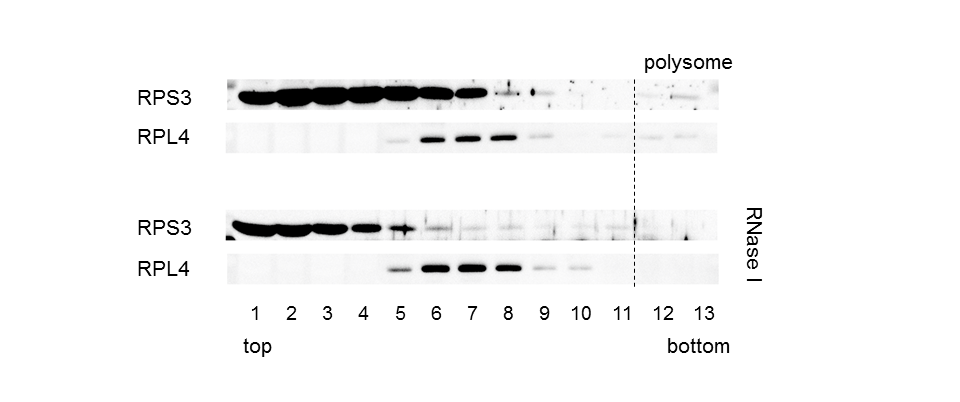


Supplemental Figure 3. The distribution of ribosomal proteins in polysome analysis. Representative ribosomal proteins (RPS3 and RPL4) on individual gradient fractions were detected by Western blot. The putative polysome fractions (fractions 12 and 13) could be converted to monosomes by RNase I treatment.
